# Supplementary material for: Computer vision syndrome and associated factors among urban and rural bankers in Trinidad and Tobago
Source: PeerJ. 2024 Dec 13;12:e18584. doi: 10.7717/peerj.18584 (PMC11648678; doi:10.7717/peerj.18584)
Supplement: Supplemental Information 2 — This questionnaire is designed to assess the prevalence and impact of Computer Vision Syndrome (CVS) among bankers. CVS is a modern-day concern arising from prolonged exposure to digital screens, which can lead to various ocular discomforts and vision-related issues. [file peerj-12-18584-s002.docx]

***Computer Vision Syndrome Questionnaire ( Bankers)***

1. **What is your gender?**

Male

Female

1. **How old are you?**

18-23

|23-28

| 28-33

| 33-38

| 38-43

| 43-48

| 48-53

| 53-58

| 58-63

1. **What is your ethnic background?**

African

|Caucasian

| East Indian

|Asian

| Syrian

|Hispanic

|Mixed

1. **What area is your bank located**?

__________________________

1. **Is your bank located in a rural or urban area ?**

Urban

| Rural

| Other:

1. **What is your highest education level that you have completed?**

CSEC level

| CAPE level

|Bachelor’s degree

|Master’s degree

|PhD

1. **How would you describe the seating position at the work place?**

Appropriate | Inappropriate

1. **What type of device do you most use while on the job ?**

Laptop

| Desktops

| Tablet

| Cellular Phone

1. **While doing computer work, is the top of the screen :**

Below eye level?

| Above eye level?

| At eye level

1. **How many hours do you spend using a computer daily?**

>6 hours

| ≤6 hours

1. **How long has it been since you started using computers regularly.**

>6 hours

| ≤6 hours

1. **Do you take breaks while using the computer during the day, if so how long do these breaks last?**

>20 minutes

| ≤20 minutes

| None at all

1. **Do you wear eyeglasses?**

Yes

| No

1. **If answer to the previous question is yes is it covered with:**

Anti reflective coating?

| Blue light blockers?

1. **Do you blink frequently while using the computer?**

Yes

| No

1. **How will you describe the source of lighting in the office?**

Natural light

| Fluorescent light

1. **Do you adjust the computer brightness to an appropriate intensity? Or, do you use it as is?**

I adjust computer brightness

| No i do not adjust the computer’s brightness

1. **Do you experience glare on your display screen?**

Yes

| No

1. **Do you use anti-glare coatings on computers? or spectacles?**

Yes

| No

1. **Do you have any systematic diseases such as diabetes, hypertension or thyroids?**

Yes

| No

Respond true or false to the following questions using your current knowledge

1. **There are eye problems caused by excessive and prolonged computer use.**

True

| False

1. **Lights from computers can affect the eye.**

True

| False

1. **Adjusting the brightness and contrast of a computer is useful.**

True

| False

1. **There are materials that can decrease glare from computers.**

True

| False

1. **There are spectacles made to protect your eyes from the light emitted from computers.** True

| False

1. **One should always take a break in between computer use.**

True

| False

1. **Computer-related eye problems cannot be resolved medically.**

True

| False

1. **Computers are made in such a way as not to cause harm to the human eye.**

True

| False

Ocular complaints, for each of the eye complaints listed state whether you experience them by saying yes or no, If yes then rate the severity from 1-10.

1. **Blurred Vision**

**Yes**

**No**

**If yes, Severity: 1 2 3 4 5 6 7 8 9 10**

1. **Redness of Eyes**

**Yes**

**No**

**If yes, Severity: 1 2 3 4 5 6 7 8 9 10**

1. **Headaches**

**Yes**

**No**

**If yes, Severity: 1 2 3 4 5 6 7 8 9 10**

1. **Dry Eyes**

**Yes**

**No**

**If yes, Severity: 1 2 3 4 5 6 7 8 9 10**

1. **Eye Fatigue**

**Yes**

**No**

**If yes, Severity: 1 2 3 4 5 6 7 8 9 10**

1. **Burning Sensation of Eyes**

**Yes**

**No**

**If yes, Severity: 1 2 3 4 5 6 7 8 9 10**

1. **Watery Eyes**

**Yes**

**No**

**If yes, Severity: 1 2 3 4 5 6 7 8 9 10**

1. **Double Vision**

**Yes**

**No**

**If yes, Severity: 1 2 3 4 5 6 7 8 9 10**

1. **Did you make effort to resolve your ocular compliant?**

Yes

No

1. **If yes, What measures did you take to resolve your ocular complaint? If other please explain briefly**

Take breaks

| Drink water

| Use eye drops

| Visit optometrist/ophthalmologist

| Obtain Spectacles

|Other:

1. How would you rate your quality of life using your computer to work?

1 2 3 4 5 6 7 8 9 10
