# Supplementary material for: Computer vision syndrome and associated factors among urban and rural bankers in Trinidad and Tobago
Source: PeerJ. 2024 Dec 13;12:e18584. doi: 10.7717/peerj.18584 (PMC11648678; doi:10.7717/peerj.18584)
Supplement: Supplemental Information 3 [file peerj-12-18584-s003.docx]

STROBE Statement—checklist of items that should be included in reports of observational studies

|  | Item No. | Recommendation | Page  No. | Relevant text from manuscript |
| --- | --- | --- | --- | --- |
| **Title and abstract** | 1 | (*a*) Indicate the study’s design with a commonly used term in the title or the abstract | 1&2 | Computer vision syndrome and associated factors …. |
|  |  | (*b*) Provide in the abstract an informative and balanced summary of what was done and what was found |  | Background: The modern workplace…  Methods and materials: A cross-sectional design..  Results:… the total participants, 277(74.7%) had computer vision syndrome. working in rural areas (AOR= 2.69; 95%CI 1.41- 5.13) and using eye sight glasses (AOR= 0.57; 95%CI 0.33- 0.97) was associated with computer vision syndrome |
| Introduction | | | |  |
| Background/rationale | 2 | Explain the scientific background and rationale for the investigation being reported | 3 | Studies have showed that using computers for three hours per day increases the risk of development of CVS as compared to people who use computers for less than three hours per day .Furthermore studies have found that computer users who apply anti-glare filters and eyeglass coatings over their screens complain less about ocular problems like CVS. CVS is a serious occupational hazard that needs to be addressed to ensure that the usage of computers in the digital era do not diminish the quality of life of users. So this research was aimed to assess the prevalence of computer vision syndrome and associated factors among urban and rural bankers in digitally driven modern banking landscape in Trinidad and Tobago |
| Objectives | 3 | State specific objectives, including any prespecified hypotheses | 4 | To determine the prevalence of computer vision syndrome among urban and rural bankers in digitally driven modern banking landscape in Trinidad and Tobago\  To identify factors associated with computer vision syndrome among urban and rural bankers in digitally driven modern banking landscape in Trinidad and Tobago |
| Methods | | | |  |
| Study design | 4 | Present key elements of study design early in the paper | 5 | Study Design, Setting and Period  The study utilized a cross-sectional study design,….. |
| Setting | 5 | Describe the setting, locations, and relevant dates, including periods of recruitment, exposure, follow-up, and data collection |  | Trinidad, one of the twin-islands making the country of Trinidad and Tobago. The country boasts a population of about 1.4 million as of 2019, encompassing various ethnic background. The study conducted between April and July …. |
| Participants | 6 | (*a*) *Cohort study*—Give the eligibility criteria, and the sources and methods of selection of participants. Describe methods of follow-up  *Case-control study*—Give the eligibility criteria, and the sources and methods of case ascertainment and control selection. Give the rationale for the choice of cases and controls  *Cross-sectional study*—Give the eligibility criteria, and the sources and methods of selection of participants | 5 | Inclusion and exclusion criteria  The inclusion criteria for the selected staff members were: - having at least one year of working experience, exposure to excessive screen used for over six months, and availability during the data collection period. The bankers who had a history of acute or chronic eye diseases were excluded from this study. |
|  |  | (*b*) *Cohort study*—For matched studies, give matching criteria and number of exposed and unexposed  *Case-control study*—For matched studies, give matching criteria and the number of controls per case |  |  |
| Variables | 7 | Clearly define all outcomes, exposures, predictors, potential confounders, and effect modifiers. Give diagnostic criteria, if applicable | 5 | Variables of the study  Dependent variable  Computer vision syndrome Independent variables  \  Social and demographic Questions  Personal Characteristics, Behavioral Factors, and screen use factors  knowledge |
| Data sources/ measurement | 8* | For each variable of interest, give sources of data and details of methods of assessment (measurement). Describe comparability of assessment methods if there is more than one group | 6 | A self-administered structured questionnaires was been utilized as the data collection tool. The questionnaire was carefully designed based on the analysis of existing literature |
| Bias | 9 | Describe any efforts to address potential sources of bias | 6 | The questionnaire was initially developed in English, and its form and structure were reviewed by experts to ensure clarity and comprehensibility. Expert advice was incorporated to improve the questionnaire's quality and enhance respondent understanding |
| Study size | 10 | Explain how the study size was arrived at | 5 | The sample size was determined by using a single population proportion formula. The following assumptions were applied: p, the prevalence of CVS 78.8%[13]; d is the expected margin of error (4%), Z, the standard score corresponding to a 95% confidence interval, and α, the risk of rejecting the null hypothesis (0.05). The final sample size will be 399. Participants were selected using random sampling methods. |

Continued on next page

| Quantitative variables | 11 | Explain how quantitative variables were handled in the analyses. If applicable, describe which groupings were chosen and why | 6 | … Descriptive statistics were carried out and presented with tables. Bivariable logistic regression analysis was performed to identify candidate variables for multivariable logistic regression at a P-value of 0.25 and statistically significant associations were declared at p-value < 0.05. |
| --- | --- | --- | --- | --- |
| Statistical methods | 12 | (*a*) Describe all statistical methods, including those used to control for confounding | 6 | Variance inflation factor (VIF) and tolerance were checked for multicollinearity, whose values were ≥0.1 and <10, respectively, to control confounders. |
|  |  | (*b*) Describe any methods used to examine subgroups and interactions 6 |  | Bivariable logistic regression analysis was performed to identify candidate variables for multivariable logistic regression at a P-value of 0.25 and statistically significant associations were declared at p-value < 0.05. |
|  |  | (*c*) Explain how missing data were addressed |  |  |
|  |  | (*d*) *Cohort study*—If applicable, explain how loss to follow-up was addressed  *Case-control study*—If applicable, explain how matching of cases and controls was addressed  *Cross-sectional study*—If applicable, describe analytical methods taking account of sampling strategy | 6 | The collected data was entered into excel and then exported to SPSS version 26 for analysis. The data were cleaned and checked for any missing outliers and inconsistencies before analysis. |
|  |  | (*e*) Describe any sensitivity analyses |  | N/A |
| Results | | | | |
| Participants | 13* | (a) Report numbers of individuals at each stage of study—eg numbers potentially eligible, examined for eligibility, confirmed eligible, included in the study, completing follow-up, and analysed | 7 | A total of 371 participants enrolled in this study, leading to a response rate of 92.9%. A majority of the respondents were female 235(63.3%), and 83(22.3%) of the participants are aged greater than or equal to 44 years…. |
|  |  | (b) Give reasons for non-participation at each stage |  |  |
|  |  | (c) Consider use of a flow diagram |  |  |
| Descriptive data | 14* | (a) Give characteristics of study participants (eg demographic, clinical, social) and information on exposures and potential confounders | 7 | …A majority of the respondents were female 235(63.3%), and 83(22.3%) of the participants are aged greater than or equal to 44 years. The majority of the respondents were of African Ethnicity 152(40.9%). Moreover, near to three-fourth 274(73.9%) and less than half 145(39.1%) of participants urban dwellers and had educational level of bachelor’s degree (Table 1). |
|  |  | (b) Indicate number of participants with missing data for each variable of interest | N/A |  |
|  |  | (c) *Cohort study*—Summarise follow-up time (eg, average and total amount) |  |  |
| Outcome data | 15* | *Cohort study*—Report numbers of outcome events or summary measures over time |  |  |
|  |  | *Case-control study—*Report numbers in each exposure category, or summary measures of exposure |  |  |
|  |  | *Cross-sectional study—*Report numbers of outcome events or summary measures | *7* | *According to this study around three-fourth 277(74.7%) of bankers had developed computer vision syndrome.* |
| Main results | 16 | (*a*) Give unadjusted estimates and, if applicable, confounder-adjusted estimates and their precision (eg, 95% confidence interval). Make clear which confounders were adjusted for and why they were included |  | N/A |
|  |  | (*b*) Report category boundaries when continuous variables were categorized |  |  |
|  |  | (*c*) If relevant, consider translating estimates of relative risk into absolute risk for a meaningful time period |  |  |

Continued on next page

| Other analyses | 17 | Report other analyses done—eg analyses of subgroups and interactions, and sensitivity analyses | N/A |  |
| --- | --- | --- | --- | --- |
| Discussion | | | | |
| Key results | 18 | Summarise key results with reference to study objectives | 9 | In Trinidad, the banking sector employees do not give much preference to their health and safety, as the workplace environment has poor ergonomic and safety arrangements. The occurrence of strained eyes and CVS is, therefore, a frequent occurrence. In this study 74.7% of bankers had computer vision syndrome; |
| Limitations | 19 | Discuss limitations of the study, taking into account sources of potential bias or imprecision. Discuss both direction and magnitude of any potential bias | 11 | First, the study was based on a large sample. It hence did not include any clinical examination, only self-reported data. Moreover, the use of a cross-sectional design makes it difficult for the researcher to make a clear decision regarding the relation between computer vision syndrome and various factors…. |
| Interpretation | 20 | Give a cautious overall interpretation of results considering objectives, limitations, multiplicity of analyses, results from similar studies, and other relevant evidence | 10 | Despite being easily preventable, computer vision syndrome is prevalent among bankers in Trinidad. The use of eye sight glasses and working area is significantly associated with this condition. Therefore, it is necessary to improve workplace practices by encouraging the use of anti-glare screens and anti-glare glasses for employees who work long hours on the computer. Bankers should also take regular breaks between work to reduce eye strain, and workplaces should be optimally illuminated to lessen the burden on the eyes. |
| Generalisability | 21 | Discuss the generalisability (external validity) of the study results |  |  |
| Other information | |  | | |
| Funding | 22 | Give the source of funding and the role of the funders for the present study and, if applicable, for the original study on which the present article is based | 12 | No funding |

*Give information separately for cases and controls in case-control studies and, if applicable, for exposed and unexposed groups in cohort and cross-sectional studies.

**Note:** An Explanation and Elaboration article discusses each checklist item and gives methodological background and published examples of transparent reporting. The STROBE checklist is best used in conjunction with this article (freely available on the Web sites of PLoS Medicine at http://www.plosmedicine.org/, Annals of Internal Medicine at http://www.annals.org/, and Epidemiology at http://www.epidem.com/). Information on the STROBE Initiative is available at www.strobe-statement.org.
